# Improving the communication of uncertainty in food safety on social media: a content analysis of aspartame risk messages

**DOI:** 10.3389/fpubh.2025.1636789

**Published:** 2026-01-05

**Authors:** Yanjie Chen, Shujun Luo, Liu Xu, Meng Tan

**Affiliations:** 1 Faculty of Humanities and Arts, Macau University of Science and Technology, Macau, China; 2 Media Art Research Center, Jiangxi Institute of Fashion Technology, Nanchang, China; 3 School of Sociology and Humanities, Jiangxi University of Finance and Economics, Nanchang, China; 4 Faculty of Hospitality and Tourism Management, Macau University of Science and Technology, Macau, China

**Keywords:** risk society, health communication, uncertainty frames, persuasive strategies, food safety, social media

## Abstract

**Background:**

Although social media has become a primary platform for the public to access and disseminate risk information, research on how to effectively communicate food safety uncertainty on these platforms remains limited.

**Objective:**

To explore the impact of uncertainty frames, information sources, and persuasive strategies on public engagement, we conducted a case study of aspartame risk communication on Weibo.

**Methods:**

Content analysis was conducted on 1,863 Weibo posts related to aspartame. Uncertainty frames, information sources, and persuasive strategies were manually coded. Public engagement was measured by retweets and likes, and descriptive statistics, chi-square tests, and negative binomial regression were conducted.

**Results:**

Results revealed a lack of professional voices, with only 23.0% of posts originating from professionals and medical institutions. Messages predominantly relied on the technical uncertainty frame (37.6%), which significantly diminished engagement (*p* < 0.001). However, when combined with persuasive strategies such as anecdotal evidence (*p* < 0.001) or cues to action (*p* < 0.001), this frame enhanced engagement. Moreover, appeals to authority generally reduced engagement (*p* < 0.001), whereas cues to action tended to enhance engagement across various uncertainty frames (*p <* 0.001). Hard science contributed to higher engagement only in the context of consensus uncertainty (*p* < 0.001). Anecdotal evidence and fear appeals exhibited more complex effects on engagement.

**Conclusion:**

The findings suggested that food safety risk communication on Weibo requires greater involvement of expert voices. When framing aspartame risks under different types of uncertainty, communicators should carefully select appropriate persuasive strategies to improve communication effectiveness.

## 1 Introduction

According to Ulrich Beck’s risk society theory, as humanity enters industrialized production societies, various risks and uncertainties increase significantly (1). Compared to natural risks, anthropogenic risks have a more widespread and profound impact on people’s daily lives (2). The World Health Organization (WHO) estimates that one-tenth of the global population is affected by foodborne diseases annually, with approximately 1.6 million people falling ill each day due to unsafe food (3). Food additives are considered one of the most crucial factors in this regard, presenting potential risks to human health (4). For instance, aspartame, an artificial non-sugar sweetener, is widely used in food and beverage manufacturing. The Food and Drug Administration (FDA) approved its use in 1974; however, on 14 July 2023, the International Agency for Research on Cancer (IARC) classified it as a possible carcinogen. This indicates that although aspartame is a common additive in everyday consumption, its safety remains uncertain.

Existing research has examined the dissemination of aspartame-related information through various media channels. For example, Gesualdo (5) reported that after aspartame was classified as a possible carcinogen, Google searches rose significantly and the topic remained popular on TikTok. However, widespread misinformation and disinformation about aspartame on these platforms pose serious challenges to public understanding. Grasso (6) analyzed newspapers across countries and found that differing narratives of aspartame risk further complicated consumers’ ability to distinguish facts from opinions in public health debates. Moreover, Batan et al. (7) highlighted the complex factors influencing individual risk perception and noted that social media data, such as Twitter, can provide insights into how people make decisions under uncertainty regarding sweetener safety. Although controversies over aspartame risks have attracted widespread attention, research on how to effectively communicate aspartame risk uncertainty remains limited. Previous research indicated that societal definitions of what constitutes health are socially and ideologically complex, imbued with moral, political, and economic values (8, 9). For instance, Moura and Aschemann-Witzel (8) found that French and Danish parents expressed different attitudes toward sugar consumption in their discourse on Facebook and blogs. Therefore, this study focuses on the Chinese social media platform Weibo, aiming to offer supplementary evidence for more effective communication of aspartame-related risk information. Despite its stringent censorship mechanisms, Weibo—with its large user base, high level of interactivity, and diverse participation—has become a key component of China’s public discourse ecosystem. It can effectively reflect the attitudes and viewpoints of various groups on relevant issues (10, 11). China currently has the largest number of obese individuals in the world, with over 50% of adults classified as overweight or obese (12, 13). To promote weight management and prevent related health problems, the Chinese government has outlined the “Three Reductions” policy—reducing salt, oil, and sugar—within the *Healthy China 2030 Planning Outline.* One of the key measures under the “reduce sugar” initiative is the substitution of traditional sugar with non-sugar sweeteners in everyday diets (13, 14). While non-sugar sweeteners meet consumer demand for low-sugar, low-calorie diets and help to mitigate health risks associated with excessive sugar consumption, increasing controversy over the safety of widely used sweeteners—such as aspartame, sucralose, and erythritol—has heightened public concern and skepticism regarding their broad inclusion in daily diets.

Uncertainties in food safety arising from advances in science and technology may erode public trust in societal and governmental decision-making and reduce the public’s capacity to make informed health decisions. While social media has become a crucial platform through which the public engages with and disseminates risk information, research on how to communicate food safety uncertainty effectively on such platforms remains limited (15). To address this gap, we examined the adoption of uncertainty frames, the involvement of various sources, and the variation in frame usage across different sources. Previous studies on uncertainty have primarily focused on persuasive strategies within binary uncertainty dimensions (e.g., presence/absence or degree of uncertainty), often overlooking the assessment of these strategies across distinct uncertainty frames—namely ontological, epistemic, consensus, and technical uncertainty. Accordingly, our study offers an innovative analysis of the use and impact of persuasive strategies across these uncertainty frames. Furthermore, as Weibo enables user feedback via retweets and likes, we explored how both uncertainty frames and persuasive strategies influenced public engagement. Overall, the main objective of this study is to provide a comprehensive understanding of how aspartame-related risks are communicated on Weibo, with a focus on the use of uncertainty frames, the roles of different information sources, the persuasive strategies employed, and their effects on public engagement.

## 2 Literature review

### 2.1 Uncertainty frames

According to uncertainty management theory (UMT), individuals’ perception of uncertainty is influenced by multiple factors, such as cognition, emotion, trust, social norms, and culture (16). This implies that public perceptions of food risks depend not only on food technologies but also on social construction processes. How governments, the media, and scientists communicate uncertainty strongly influences how people perceive these risks and make health decisions (17, 18). Prior research has shown that science communication often employs ontological, epistemic, consensus, and technical uncertainty frames to convey the uncertainties inherent in scientific research (19, 20). From an ontological perspective, food safety risks are viewed as inherent to food, arising from food technology or scientific research. Given the limits of scientific knowledge and the complexity of the issue, such risks are difficult to fully predict or eliminate. Communicators therefore often stress the inevitability of risk and promote precautionary principles and adaptive management (21). From a cognitive perspective, food safety risks are linked to the limitations and gaps in current scientific knowledge. In theory, these can be reduced through further research, more comprehensive data, advanced technologies, improved experimental designs, and larger samples (19, 22). It is important to note that both ontological and epistemic uncertainty center on knowledge but differ in perspective: the former looks forward from the present, emphasizing the variability of knowledge, while the latter looks backward, focusing on the inadequacy of existing knowledge. Unlike the ontological and epistemic uncertainty frames, the consensus uncertainty frame attributes food safety risks to differing viewpoints or conflicting evidence (22). For instance, regarding aspartame, some studies found it to be safe within acceptable intake levels, while others suggested potential associations with liver cancer, breast cancer, and other health risks (23, 24). Uncertainty in food safety is also seen as a byproduct of scientific pursuits in technology, industry, and social progress (2). Due to the complex technical issues and potential risks involved, which are often difficult for laypeople to understand, scientists regularly present scientific results using ranges, probabilities, or confidence intervals. This approach, termed technical uncertainty, involves quantifying risks to help the public intuitively assess their magnitude and establishes acceptable risk thresholds to facilitate societal acceptance (25, 26).

In summary, uncertainty in food safety can be categorized into ontological, epistemic, consensus, and technical types. These distinct uncertainty frames reflect different perspectives on food risk and typically correspond to varying approaches to health responses. However, existing studies have primarily examined the impact of different types of uncertainty frames on public behavior under experimental conditions (19, 27, 28), with few studies employing content analysis to assess the application of these frames in the context of food safety risk communication via social media. Analyzing the use of different uncertainty frames helps reveal societal tendencies in constructing food safety issues and identifies problems in current communication practices regarding food safety uncertainties.

### 2.2 Sources

In general, laypeople cannot evaluate risk information based solely on media content; the information source often serves as an important supplementary cue (29). Journalists adhering to high-quality news standards are expected to objectively report the limitations of existing research or conflicting scientific views (30). However, in practice, journalists, as intermediaries between experts and the public, not only rely on expert evidence but also adjust the framing of information based on organizational objectives, personal understanding, and audience expectations (31). Therefore, the media may optimize or omit uncertainties when disseminating risk information, resulting in a gap between perceived and actual risk levels (32). Furthermore, to improve the quality and credibility of risk communication on social media, countries worldwide have been promoting the involvement of professionals and institutions. However, previous studies have shown that their participation in social media remains low (11, 33). This is partly because they have fewer followers than celebrities or social media influencers, especially among less-educated populations (34, 35). Besides, they often face the challenge of balancing information transparency with maintaining public trust, which also affects their willingness to engage in communicating uncertainty on social media (36, 37).

In addition, social media influencers (SMIs) have increasingly played a role in health risk communication in recent years (38). SMIs are microcelebrities who gain large followings and recognition through social media content, unlike traditional celebrities, such as actors or models, who achieve fame through their professions or performances (39). Although experts can be seen as influencers to some extent, they mainly rely on their expertise, while SMIs often combine knowledge, experience, and values into personal narratives when communicating risks (40). Prior studies have recognized the contribution of SMIs to risk communication, highlighting their ability to promote public engagement and influence public understanding of risks (41). However, few studies have specifically examined the extent of SMIs’ involvement in food safety risk communication, how they handle uncertainty, or how their approach differs from that of the media, professionals, and institutions. Our study focuses on information from these three types of sources, which constitute the primary sources for the public’s risk assessment. By analyzing their level of involvement and the uncertainty frames they adopt, the aim is to assess their influence, strategic differences, and positions in food safety risk communication on social media. Thus, we propose RQ1, RQ2, and RQ3 (see Table 1).

**Table 1:** Research questions and analytical methods applied.

Research question	Analytical method
RQ1: To what extent are different types of uncertainty frames employed in the communication of aspartame-related risks on Weibo?	Descriptive analysis
RQ2: To what extent do different information sources contribute to the communication of aspartame-related risks on Weibo?	Descriptive analysis
RQ3: Are there differences in the types of uncertainty frames adopted by different sources when describing aspartame-related information?	Chi-square (*χ*^2^) test
RQ4: What persuasive strategies are employed in aspartame-related information on Weibo?	Descriptive analysis
RQ5: Are there differences in the persuasive strategies between different sources?	Chi-square (*χ*^2^) test
RQ6: Are there differences in the persuasive strategies across different types of uncertainty frames in aspartame-related information on Weibo?	Chi-square (*χ*^2^) test
RQ7: How do different types of uncertainty frames affect public engagement?	Negative binomial regression
RQ8: How does the impact of persuasive strategies on public engagement differ across different types of uncertainty frames?	Negative binomial regression

### 2.3 Persuasive strategies

Uncertainty communication may lead to confusion, discomfort, or skepticism regarding the credibility of the communicator, especially when the situation is ambiguous, the information is inconsistent, or definitive conclusions are lacking (16). Thus, when communicators choose to disclose uncertainty, they often employ persuasive strategies to enhance the credibility of the information. Scholars have investigated the application of various persuasive strategies in uncertainty communication, such as appeals to authority (42), emphasizing perceived susceptibility and severity (43), framing in terms of gains or losses (44), and providing specific action recommendations (45). However, these studies primarily focus on the binary dimensions of uncertainty, such as its presence or absence, or high or low levels, and give limited attention to analyzing the use and impact of persuasive strategies within different uncertainty frames, such as epistemic, consensus, technical, and ontological uncertainty. Gustafson and Rice (19, 20) demonstrated that these different types of uncertainty frames can affect the public’s beliefs, perceived credibility, and behavioral intentions, leading to positive, negative, or neutral outcomes. Based on this, our study hypothesizes that due to the influence of uncertainty frames on how audiences perceive risks and process information, persuasive strategies may vary in their effectiveness across different framing conditions. This means that risk communicators should consider frame-specific strategies to optimize communication outcomes.

The Elaboration Likelihood Model (ELM) is a key theory in the study of persuasive communication. It considers two potential processing routes—central and peripheral—that the public may rely on during information processing (46). The central route refers to individuals processing information in a detailed and systematic manner, while the peripheral route involves making quick judgments based on simple cues or features of the information (47). Based on previous research, we treat hard science as a systematic cue. Hard science messages typically reference journal articles or empirical studies, providing concrete data and findings to help the public better understand complex issues (48). Additionally, we treat anecdotal evidence and appeals to authority as heuristic cues. Anecdotal evidence involves using personal or third-party experiences and narratives to support a particular claim (49). Appeals to authority means using viewpoints or recommendations from authoritative institutions, experts, governments, or other credible individuals to support a claim (50). We categorize fear appeals and cues to action as a combination cue, which can be either systematic or heuristic. Fear appeals are designed to evoke fear, encouraging preventive actions and self-protective behaviors. The type of cue used depends on whether the message triggers danger control (based on cognitive processing) or fear control (based on emotional response) (51). Cues to action involve receiving prompts (from mass media, advice from professionals or significant others) that encourage individuals to take action (52). The type of cue depends on whether the recommendation is straightforward and clear or complex. Our study explores how persuasive strategies are employed in communicating aspartame-related risks on Weibo and accordingly proposes RQ4, RQ5, and RQ6.

In addition, we measure the impact of different types of uncertainty frames on public engagement through retweets and likes. Based on previous research, the public can express interest, perceived usefulness, positive attitudes, and agreement with viewpoints by liking content (53). Meanwhile, they can also choose to retweet the post, which not only demonstrates their interest in the issue but also shows their willingness to share this information with others and engage in in-depth discussions and interactions (54). Although retweeting does not always signal agreement, it serves as an indicator of public interest in the issue and promotes dialogue and understanding (48, 55). Since different types of uncertainty frames may influence individuals’ risk perceptions to different extents, we raise RQ7. Additionally, we also want to examine whether persuasive strategies lead to different effects on public engagement across different types of uncertainty frames, and raise RQ8.

## 3 Methods

### 3.1 Sample selection

A quantitative content analysis was conducted using Weibo posts containing the keywords “aspartame,” “non-sugar sweeteners,” “artificial sweeteners,” or “sugar substitute” as the data source. Weibo was selected for its wide reach and influence in China. The sampling period spanned from 1 May 2023 to 30 April 2024. During this time, the WHO issued updated guidance on non-sugar sweeteners (NSS) on 15 May 2023, advising against their use for weight management or the prevention of non-communicable diseases (NCDs). On 14 July 2023, the WHO further announced that aspartame could be carcinogenic.

**Figure 1:**
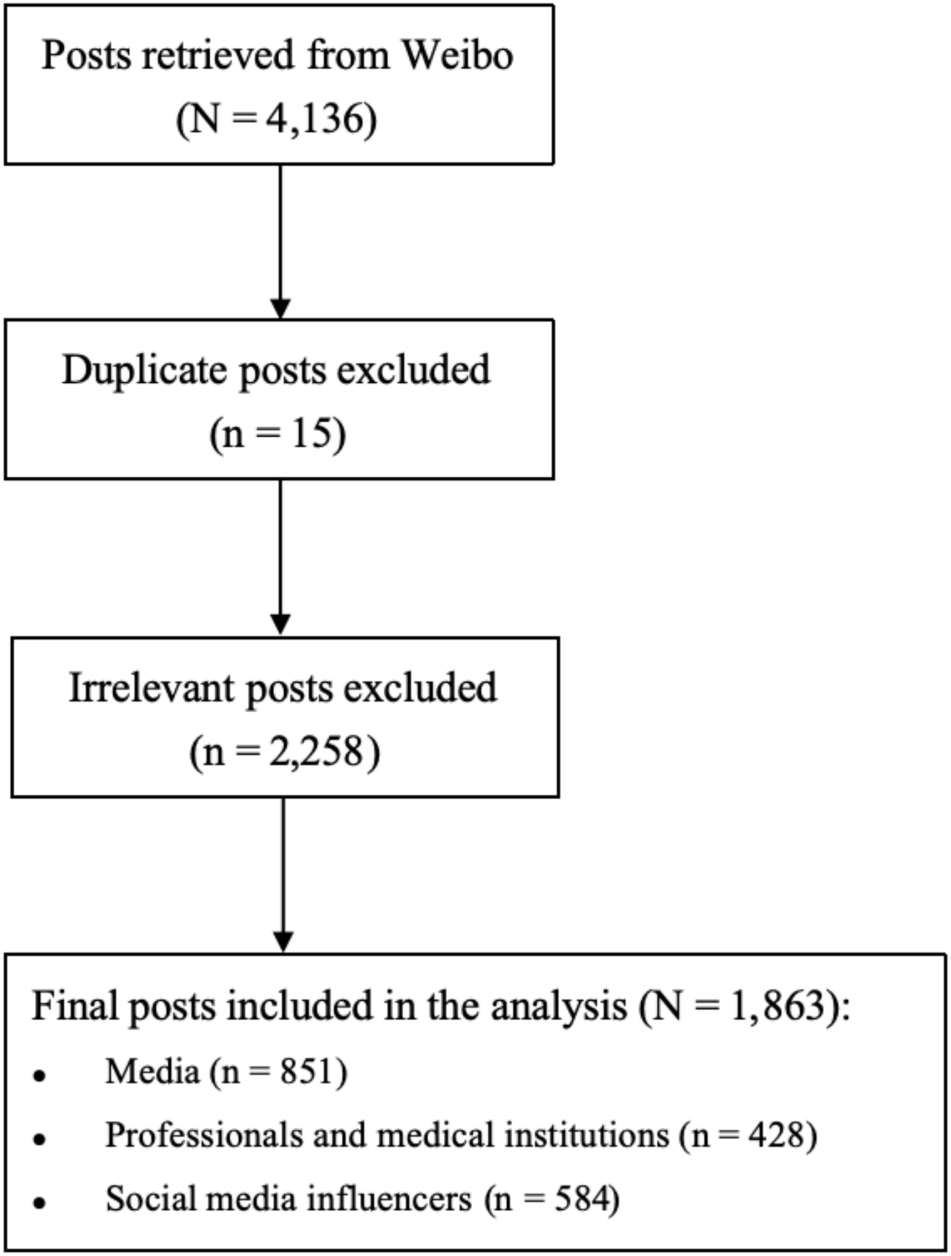
Data cleaning and selection process of Weibo posts.

A total of 4,136 Weibo posts were retrieved, including plain text as well as posts containing images, videos, or external links. After removing 15 duplicates and excluding 2,258 posts identified as advertisements, irrelevant to aspartame, or from sources outside the scope of this study, 1,863 posts remained for analysis. The final dataset comprised 851 posts from media outlets (both official and internet-based), 428 posts from professionals and medical institutions, and 584 posts from social media influencers (SMIs). The data collection flow is shown in Figure 1.

### 3.2 Coding procedure

We coded uncertainty frames (epistemic uncertainty, consensus uncertainty, technical uncertainty, ontological uncertainty and no uncertainty), persuasive strategies (hard science, anecdotal evidence, appeals to authority, fear appeals and cues to action) and sources (media, professionals and medical institutions, and social media influencers).

For uncertainty frames, we employed a dominant category approach, whereby the frame most frequently reflected in a post’s sentences was designated as the dominant frame. For example, if a post contained 2 sentences reflecting epistemic uncertainty, 3 sentences reflecting consensus uncertainty, and 5 sentences reflecting technical uncertainty, the post was classified as exhibiting technical uncertainty. In cases where the number of sentences was equal across frame types, the primary coder used the post’s hashtags, topic sentences, and broader context to determine the dominant frame. Each frame was then coded as 1 if dominant and 0 if non-dominant for statistical analysis, resulting in a binary coding scheme.

For persuasive strategies, each post was assessed for the presence of one or more strategies, with each strategy coded as 1 if present and 0 if absent, resulting in a binary coding scheme. Source categories were coded as a nominal variable, with media = 1, professionals and medical institutions = 2, and social media influencers = 3.

### 3.3 Reliability test

All data were independently coded by two coders. Inter-coder reliability was assessed using a random subsample (*n* = 373; 20.02% of the dataset), with Krippendorff’s alpha (*α*) values ranging from 0.839 to 0.974 (see Table 2).

**Table 2:** Coding book.

Theme and subtheme		Inter-coder reliability (Krippendorff’s *α*)	Definition	Example
Information source	Media	0.974	Accounts of official media in China, owned by the government, are allowed to conduct news interviews, and internet-based media can edit news but are not permitted to conduct interviews	People’s daily, Xinhua daily, Toutiao news, NetEase health
	Professionals and medical institutions		Accounts of persons from medical fields or organizations providing medical service to the public	Doctors, medical and biological experts, hospitals, medical research institutions
	Social media influencers (SMIs)		Accounts of persons without a medical background but with social influence	Science bloggers, lifestyle bloggers, fashion bloggers
Uncertainty frames	Epistemic uncertainty	0.886	Lack of knowledge or unawareness, emphasizing the scarcity of existing knowledge	Aspartame has limited evidence of carcinogenicity in humans, particularly for liver cancer. The evidence of carcinogenicity found in animal experiments, as well as the potential mechanisms leading to cancer, are also limited.
	Consensus uncertainty		Disagreements among experts or stakeholders, or inherent conflicts within scientific evidence itself	The International Agency for Research on Cancer (IARC) recognizes limited evidence regarding the carcinogenicity of aspartame in humans, categorizing it as possibly carcinogenic. In contrast, the International Council of Beverages Associations (ICBA) argues that it contradicts decades of high-quality scientific evidence and that the conclusions drawn from low-quality scientific research may mislead consumers.
	Technical uncertainty		Due to measurement errors, modeling approximations, and statistical assumptions, it is often represented using probabilities, ranges, and confidence intervals	Aspartame can increase the risk of cancer by 15%, with the highest increases in the risk of breast cancer and obesity-related cancers reaching up to 22 and 15%, respectively.
	Ontological uncertainty		Scientific knowledge is always temporary, emphasizing the unknown and unknowable nature of knowledge	The WHO’s guidelines on non-sugar sweeteners are based on evidence derived from current and past clinical studies. However, this does not imply that they represent the ultimate or universally applicable truth. The debate over sugar substitutes awaits more robust scientific research to reach a conclusive end.
	No uncertainty		The information is presented clearly, accurately, and fully known, without any ambiguity, randomness, or unknown factors	The World Health Organization (WHO) has issued guidelines on non-sugar sweeteners, advising against the use of aspartame, acesulfame, saccharin, and other non-sugar sweeteners for weight control or reducing the risk of non-communicable diseases.
Persuasive strategies	Hard science	0.873	Citing journal articles or scientific research to support one’s arguments	A study published in the renowned medical journal *Nutrients* shows that, compared to individuals who do not consume sugary beverages, those who drink large amounts daily have a 60% increased risk of fatty liver disease. Additionally, those who drink sugar-free beverages have a 78% higher risk of developing fatty liver disease.
	Anecdotal evidence	0.919	Using personal or others’ experiences and stories to support a claim	Xiao Xia (a pseudonym), a young man from Zhejiang Province in China, had poor dietary habits. To lose weight, he regularly drank sugar-free beverages instead of water for an extended period, which ultimately led to the development of diabetes.
	Appeals to authority	0.902	Citing the views or recommendations of authoritative institutions, experts, or government agencies	Aspartame, one of the most common artificial sweeteners in the world, has been classified by the International Agency for Research on Cancer (IARC) as a “possibly carcinogenic to humans (Group 2B).”
	Fear appeals	0.839	Persuading people to maintain a healthy diet or avoid consumption by highlighting the potential severe consequences of aspartame intake, such as the risk of cancer	Sugary beverages (including those containing sugar substitutes like aspartame), often referred to as “happy drinks,” are more accurately described as “liver-damaging” and “kidney-damaging” drinks. The more you consume, the greater the risk of fatty liver and kidney damage.
	Cues to action	0.847	Providing specific consumption recommendations in response to the potential safety risks associated with aspartame	Assuming no other food intake, an adult weighing 70 kilograms would need to drink 9 to 14 cans, or more, of a weight-loss soft drink containing 200 to 300 milligrams of aspartame per can exceed the recommended daily intake limit. Therefore, within this daily limit, it can be consumed safely.

### 3.4 Statistical analysis

We conducted descriptive analyses to address RQ1, RQ2, and RQ4. To examine group differences in RQ3, RQ5, and RQ6, we employed chi-square (*χ*^2^) tests, one of the most widely used methods for assessing associations or differences between categorical variables (56). Since the engagement variables were over-dispersed counts, negative binomial (NB) regression models were applied to RQ7 and RQ8. NB regression is widely used in applied research to handle over-dispersed count outcomes (57, 58).

## 4 Results

In response to RQ1, we found that most Weibo posts adopted the technical uncertainty frame, which occurred most frequently (37.6%, *n* = 644), followed by the no uncertainty frame (35.2%, *n* = 603), consensus uncertainty (24.4%, *n* = 419), and epistemic uncertainty (10.8%, *n* = 185). The ontological uncertainty frame was the least prevalent (0.7%, *n* = 12). Regarding RQ2, the results showed that Weibo posts from media were the most frequent (45.7%, *n* = 851), followed by SMIs (31.3%, *n* = 584), and professionals and medical institutions (23.0%, *n* = 428). In response to RQ3, *χ*^2^ tests revealed significant differences among the different sources, *χ*^2^ = 401.86, *p* < 0.001 (see Table 3). *Post hoc* analysis indicated that media used epistemic, consensus, and technical uncertainty frames more frequently than expected by chance (*χ*^2^ test), and fewer no-uncertainty frames than expected. Professionals and medical institutions used more ontological uncertainty frames than expected. SMIs used more no uncertainty frames and fewer epistemic, consensus, and technical uncertainty frames than expected.

**Table 3:** The types of uncertainty frames used by different sources.

Uncertainty frames	Media (%)	Professionals, medical institutions (%)	SMIs (%)
Epistemic uncertainty	122 (14.3)	34 (7.9)	29 (5.0)
Consensus uncertainty	253 (29.7)	104 (24.3)	62 (10.6)
Technical uncertainty	366 (43.0)	147 (34.3)	131 (22.4)
Ontological uncertainty	2 (0.2)	8 (1.9)	2 (0.3)
No uncertainty	108 (12.8)	135 (31.6)	603 (61.7)

SMIs, Social Media Influencers.

For RQ4, the results indicate that communicators employed various persuasive strategies in conveying aspartame-related information. Specifically, appeals to authority were the most frequently used, followed by cues to action, while hard science was the least used (see Table 4).

**Table 4:** The use of persuasive strategies by different sources.

Persuasive strategies	Media (%)	Professionals, medical institutions (%)	SMIs (%)	*χ* ^2^	Total
Hard science	78 (9.2)	140 (32.7)	46 (7.9)	157.50^***^	264 (14.17)
Anecdotal evidence	212 (24.9)	17 (4.0)	162 (27.7)	98.69^***^	391 (20.99)
Appeals to authority	674 (79.2)	260 (60.7)	226 (38.7)	242.35^***^	1,160 (62.27)
Fear appeals	149 (17.5)	88 (20.6)	114 (19.5)	1.99	351 (18.84)
Cues to action	355 (41.7)	186 (43.5)	144 (24.7)	54.04^***^	685 (36.77)

SMIs, Social Media Influencers; *χ*^2^, chi-square; ^***^p < 0.001.

In response to RQ5, we found that all persuasive strategies, except for fear appeals, showed significant differences across sources (*p* < 0.001) (see Table 4). Specifically, *post hoc* analysis revealed that media relied predominantly on anecdotal evidence, appeals to authority, and cues to action, while hard science was used less frequently than expected. Professionals and medical institutions emphasized hard science and cues to action, with anecdotal evidence appearing less often than expected. SMIs tended to favor anecdotal evidence, using hard science, appeals to authority, and cues to action less frequently than expected.

In response to RQ6, the results indicate that all persuasive strategies differed significantly across uncertainty frames (*p* < 0.001) (see Table 5). Particularly, *post hoc* analyses showed that in the epistemic uncertainty frame, appeals to authority and fear appeals predominated, whereas hard science and anecdotal evidence were less frequently employed than expected. For the consensus uncertainty frame, hard science, anecdotal evidence, and appeals to authority were prominent, while fear appeals and cues to action appeared less often. Within the technical uncertainty frame, hard science, appeals to authority, and cues to action were particularly common. Conversely, in the no uncertainty frame, hard science, appeals to authority, and cues to action were less prevalent.

**Table 5:** The use of persuasive strategies across different types of uncertainty frames.

Persuasive strategies	Epistemic uncertainty (%)	Consensus uncertainty (%)	Technical uncertainty (%)	Ontological uncertainty (%)	No uncertainty (%)	*χ* ^2^	Total
Hard science	7 (3.8)	108 (25.8)	117 (18.2)	3 (25.0)	29 (4.8)	115.87^***^	264 (14.17)
Anecdotal evidence	6 (3.2)	139 (33.2)	115 (17.9)	0 (0.0)	131 (21.7)	93.40^***^	345 (18.52)
Appeals to authority	174 (94.1)	339 (80.9)	450 (69.9)	4 (33.3)	193 (32.0)	396.67^***^	1,160 (62.27)
Fear appeals	66 (35.7)	35 (8.4)	136 (21.1)	2 (16.7)	112 (18.6)	66.68^***^	351 (18.84)
Cues to action	78 (42.2)	68 (16.2)	405 (62.9)	2 (16.7)	132 (21.9)	326.82^***^	685 (36.77)

*χ*^2^, chi-square; ^***^p < 0.001.

Regarding RQ7, the model predicting retweet counts from uncertainty frames was statistically significant (*χ*^2^(4) = 155.61, *p* < 0.001) (see Table 6). With the no uncertainty frame as the reference category, the epistemic uncertainty frame significantly positively predicted retweets (*B* = 0.37, SE = 0.09, IRR = 1.45, *p* < 0.001), whereas the consensus (*B* = −0.49, SE = 0.07, IRR = 0.61, *p* < 0.001) and technical uncertainty frames (*B* = −0.45, SE = 0.06, IRR = 0.64, *p* < 0.001) significantly negatively predicted retweets. For like counts, the model was also significant (*χ*^2^(4) = 424.82, *p* < 0.001). Compared to the no uncertainty frame, the epistemic (*B* = −0.32, SE = 0.08, IRR = 0.72, *p* < 0.001), consensus (*B* = −1.40, SE = 0.06, IRR = 0.25, *p* < 0.001), and technical frames (*B* = −0.54, SE = 0.06, IRR = 0.58, *p* < 0.001) all significantly and negatively predicted the number of likes.

**Table 6:** The effect of different types of uncertainty frames on engagement.

Uncertainty frames	Retweet			Like		
	*B*	SE	IRR	*B*	SE	IRR
Epistemic uncertainty	0.37^***^	0.09	1.45	−0.32^***^	0.08	0.72
Consensus uncertainty	−0.49^***^	0.07	0.61	−1.40^***^	0.06	0.25
Technical uncertainty	−0.45^***^	0.06	0.64	−0.54^***^	0.06	0.58
Ontological uncertainty	0.20	0.30	1.22	−0.30	0.29	0.74
No uncertainty	0.00^a^	–	1.00	0.00^a^	–	1.00
Log-likelihood	−6,181.05			−9,647.80		
Model *χ*^2^	155.61^***^			424.82^***^		
df	4			4		
*N*	1,863			1,863		

^a^Note that the no uncertainty frame was used as the reference group in this analysis.

B, Regression Coefficient; SE, Standard Error; IRR, Incident Rate Ratio; ^***^p < 0.001.

In response to RQ8, the results showed that engagement decreased when hard science and appeals to authority were employed, but increased with fear appeals and cues to action within the epistemic uncertainty frame. In the consensus uncertainty frame, mentions of hard science, anecdotal evidence, and cues to action were associated with higher engagement, whereas fear appeals reduced engagement. Appeals to authority increased retweets but decreased likes. In the technical uncertainty frame, anecdotal evidence and cues to action enhanced engagement, whereas hard science, appeals to authority, and fear appeals were associated with lower engagement. In the no uncertainty frame, hard science, anecdotal evidence, appeals to authority, and cues to action were all associated with lower engagement, whereas fear appeals increased retweets but decreased likes (see Table 7).

**Table 7:** The effect of persuasive strategies on engagement across different types of uncertainty frames.

Persuasive strategies	Epistemic uncertainty		Consensus uncertainty		Technical uncertainty		No uncertainty	
	Retweet	Like	Retweet	Like	Retweet	Like	Retweet	Like
	*B*	*B*	*B*	*B*	*B*	*B*	*B*	*B*
Hard science	−0.23	−1.52^***^	1.28^***^	1.08^***^	−0.98^***^	−0.15	−0.44^*^	−1.15^***^
Anecdotal evidence	−0.40	0.08	0.23	0.84^***^	1.67^***^	2.87^***^	−0.07	−1.22^***^
Appeals to authority	−0.15	−1.05^***^	0.33^*^	−1.48^***^	−1.44^***^	−1.53^***^	−1.22^***^	−1.56^***^
Fear appeals	1.49^***^	1.39^***^	0.10	−1.71^***^	0.14	−0.53^***^	0.35^***^	−0.33^*^
Cues to action	1.86^***^	1.20^***^	1.82^***^	2.26^***^	0.44^***^	−0.07	0.01	−0.81^***^
Log-likelihood	−555.29	−874.41	−1,166.04	−1,495.30	−1,729.83	−2,672.90	−2,054.62	−3,194.80
Model *χ*^2^	330.63^***^	245.16^***^	237.51^***^	632.82^***^	539.92^***^	1,315.04^***^	151.08^***^	497.74^***^
df	5	5	5	5	5	5	5	5
N	185	185	419	419	644	644	603	603

Due to the small sample size for ontological uncertainty, the model did not fit and was therefore excluded.

B, Regression Coefficient; ^*^p < 0.05; ^***^p < 0.001.

## 5 Discussion

The advancement of science and technology is inherently accompanied by various forms of uncertainty, among which those related to the development of food technologies are more likely to arouse public concern. Based on this, it is essential to establish effective risk communication strategies to help the public identify, understand, and appropriately respond to potential risks in their decision-making processes. This study primarily explored the communication of food safety risk uncertainty on social media using data from Weibo about aspartame. The findings can help identify shortcomings in current practices and provide insights for improving the effectiveness of risk communication strategies.

This study found that aspartame-related information on Weibo was predominantly framed using the technical uncertainty perspective. The result aligns with earlier studies (25, 59, 60), suggesting that in modern industrial societies, risk decision-makers or communicators tend to use the technical uncertainty frame when communicating with lay people. Because this frame employs quantitative measures (e.g., proportion, scope), it can help the public balance risks and benefits, thereby enabling them to enjoy the benefits of new food technologies while avoiding potential risks (61). According to Bearth and Siegrist’s (62) meta-analysis, perceptions of risks and benefits constitute important drivers of consumer acceptance of different food technologies, lending further support to our finding regarding the prevalent use of the technical uncertainty frame.

In addition, our data revealed that aspartame risk communication on Weibo was dominated by media actors, with professional and medical institutions contributing the least. This finding is consistent with Li et al.’s (11) findings on HPV vaccine risk communication on Weibo, as well as with Rao et al.’s (63) study of health issues such as childhood obesity, smoking, and cancer on the platform. Taken together, these studies suggest that risk communication on Weibo may be more media-driven than led by professionals. Furthermore, we found that media actors on Weibo tend to disclose uncertainty when communicating aspartame risks, which may be due to their social supervision role and pursuit of visibility and economic gain (64, 65). In comparison, SMIs are more inclined to convey definitive messages, framing aspartame as wholly safe or wholly harmful. This observation corroborates existing scholarly concerns that SMIs, in an effort to increase persuasive impact, may oversimplify scientific information and thereby mislead the public (66, 67). Unlike media and SMIs, it was observed that professionals and medical institutions prefer the ontological uncertainty frame, indicating greater caution in food safety risk communication. According to Krebs (68), in food safety risk communication, scientists may neither endorse the media’s way of portraying uncertainty nor favor the approach adopted by vested interests.

Our study also confirmed that in Weibo communications about aspartame risks, appeals to authority were most common, whereas hard science was infrequent. A possible explanation is that, given the inherent complexity of aspartame’s uncertainty communication, which can easily confuse the public, communicators tend to rely on authoritative endorsements to reduce individuals’ cognitive burden. Previous research has also shown that users prefer concise, easily understandable heuristic content, particularly on social media (69, 70). Consequently, communicators discussing aspartame risks on Weibo tend to rely more on appeals to authority than on hard science to improve the effectiveness of their communications.

With regard to the persuasive strategies employed by different sources, our study found that media actors, as social monitors and information providers, tend to use appeals to authority to strengthen credibility and employ anecdotal evidence and cues to action to facilitate public understanding and response to aspartame risks. Professionals and medical institutions primarily employ hard science and behavioral cues to meet the demands of producing expert content, whereas SMIs mainly rely on anecdotal evidence to communicate aspartame risks, aiming to elicit emotional resonance among their followers and promote engagement. In general, different sources in Weibo communications about aspartame risks favor persuasive strategies that reflect their social roles and objectives, consistent with earlier findings regarding the communication styles of these three source categories (32, 71, 72). Moreover, we found that while communicators’ persuasive strategies varied across uncertainty frames in describing aspartame risks, appeals to authority consistently dominated, representing a novel finding in the literature on uncertainty framing.

Furthermore, this study first examined how the use of different types of uncertainty frames and persuasive strategies influence public engagement, providing novel extensions and empirical support for existing research. Specifically, we observed that although the technical uncertainty frame was the most commonly used to present aspartame information, it failed to elicit substantial public engagement on Weibo. Based on previous research, frequent risk quantification and the establishment of acceptable risk levels can potentially increase public awareness of the concept of a risk society, while reducing sensitivity to individual risks (26). Moreover, the consensus uncertainty frame was found to negatively influence both retweets and likes, consistent with prior studies reporting its adverse effects on credibility, behavioral intentions, and similar outcomes (20, 45). The negative effect may stem from the consensus uncertainty frame presenting diverse or conflicting evidence, potentially increasing public confusion and uncertainty in complex situations (45, 73). For instance, Iles et al. (45) reported that exposure to conflicting information diminishes perceived scientific consensus on recommended red meat consumption. Additionally, the epistemic uncertainty frame was found to increase retweets while reducing likes. Psychological theory suggests that acquiring knowledge about the external world is vital for individuals’ adaptive capacity, whereas insufficient knowledge can undermine their sense of control (74). This suggests that consumers are motivated to share risk-related information to alert others, but may not welcome uncertainty about aspartame’s safety.

Our findings further demonstrated that, across uncertainty frame conditions, appeals to authority generally hinder public engagement, contrary to prior studies showing that authoritative citations can alleviate the negative impacts of uncertainty in communication (75, 76). This may reflect a declining public trust in uncritically accepted scientific knowledge, as food safety issues have become increasingly prominent in recent years, with the credibility of professional authorities also coming under growing scrutiny (77, 78). By contrast, cues to action were found to generally enhance public engagement across all uncertainty frames, aligning with previous studies on their application in food safety communication (79, 80). According to the Health Belief Model and Protection Motivation Theory, the public is more inclined to accept risk information that offers clear guidance on how to act. Such information can enhance their confidence in their ability to cope with risks, thereby boosting their sense of self-efficacy and further motivating participation (52, 81). For example, Piee et al. (82) found that cues to action provided on food packaging play an important role in improving students’ food safety behaviors. Additionally, our data indicated that hard science positively influenced both retweets and likes under the consensus uncertainty frame, whereas appeals to authority had a positive effect on retweets but a negative effect on likes. This finding is consistent with Smithson (83), who reported that the public is more receptive to conflicts within scientific evidence than to disagreements among scientists or research institutions. This phenomenon can be explained by the public’s tendency to see conflicts within scientific evidence as collaborative opportunities, whereas disputes between scientists or institutions are attributed to personal gain or ineptitude, lowering trust (84, 85). Moreover, fear appeals were found to enhance both retweets and likes exclusively within the epistemic uncertainty frame. This suggested that fear appeals may amplify individuals’ risk perception in situations of knowledge deficiency, triggering a sense of threat and urgency among the public (51). To obtain more relevant information or to raise awareness of the risk, the public may be inclined to retweet or like the content. Finally, our results showed that anecdotal evidence enhanced engagement under both the consensus and technical uncertainty frames. This may indicate that anecdotal evidence, by being concrete and vivid, could both reduce distrust from conflicting views and make complex technical information more accessible to daily-life contexts, lowering comprehension barriers.

### 5.1 Implications

First, we found that although SMIs are playing an increasingly prominent role in food safety risk communication on social media, they often rely on no-uncertainty frames, which may oversimplify the complexity of food technologies and heighten the risk of misinformation. Meanwhile, the limited participation of professionals and institutions may undermine the credibility and quality of information, allowing non-expert sources to dominate the discourse. Therefore, to improve the food safety risk communication environment on social media, it is essential to provide science literacy training for SMIs and to actively promote broader participation from professionals. In addition, the study found that the carcinogenic risk of aspartame was predominantly framed using technical uncertainty, which did not attract public engagement. A possible solution may lie in the combined use of multiple uncertainty frames. However, this study did not examine whether different combinations of uncertainty frames produce varying effects, which warrants further investigation in future research. Nonetheless, our study showed that persuasive strategies moderated the relationship between different types of uncertainty frames and public engagement. The technical uncertainty frame increased engagement when paired with anecdotal evidence or cues to action. Appeals to authority generally had negative effects, while cues to action had positive ones. Some strategies were effective only within specific uncertainty frames, for instance, hard science had a significant positive effect only when used within the consensus uncertainty frame. These findings imply that to enhance communication effectiveness, risk communicators should carefully consider their choice of persuasive strategies when employing different types of uncertainty frames to convey food safety risks. Finally, it is worth noting that previous studies have highlighted the importance of expert sources as key heuristic cues relied upon by the public in risk decision-making (86). In persuasive communication, citing expert sources is typically considered a key means of enhancing message credibility. However, this study found that appeals to authority, the most frequently employed persuasive strategy, had a predominantly negative effect on engagement in food safety risk communication on social media. This may suggest that in current food safety communication, relying on authority is no longer effective, as public trust in authority continues to decline. Future research could further explore the effectiveness of combining appeals to authority with other persuasive strategies or communication contexts.

### 5.2 Limitations

This study has several limitations. First, our study focused solely on aspartame to examine social media communication of food safety risk uncertainty. Future studies could extend this work by comparing the effects of risk uncertainty across different food processing technologies on public cognition, affect, and behavior, providing a more comprehensive understanding. Second, our research concentrated on Weibo. Subsequent studies could explore multiple social media platforms to assess how differences in audience demographics, content formats, and engagement mechanisms affect public understanding of aspartame risks. Third, our study was confined to China. Future investigations could analyze different countries or regions to better understand how political, cultural, and societal factors shape individual risk perceptions.

## 6 Conclusion

This study analyzed Weibo posts concerning aspartame to examine social media communication of food safety risk uncertainty. The findings underscored the need for greater expert involvement in food safety risk communication on Weibo. Careful consideration of persuasive strategies was warranted when communicating aspartame risks across different uncertainty frames. Particularly, we found that the most frequently used technical uncertainty frame only positively influenced user engagement on Weibo when combined with anecdotal evidence and cues to action. In addition, the negative effect of appeals to authority highlighted the need to restore the credibility of authoritative sources in Weibo risk communication to enhance public trust and engagement.

## Data Availability

The original contributions presented in the study are included in the article/supplementary material, further inquiries can be directed to the corresponding author.
